# Exploring Future Pandemic Preparedness Through the Development of Preventive Vaccine Platforms and the Key Roles of International Organizations in a Global Health Crisis

**DOI:** 10.3390/vaccines13010056

**Published:** 2025-01-10

**Authors:** Jihee Jeon, Eunyoung Kim

**Affiliations:** 1 Pharmaceutical Regulatory Affairs, Department of Pharmaceutical Industry, Graduate School, Chung-Ang University, Seoul 06974, Republic of Korea; henjjh930@cau.ac.kr; 2 Central Research Center of Epigenome Based Platform and Its Application for Drug Development, College of Pharmacy, Chung-Ang University, Seoul 06974, Republic of Korea; 3 Data Science, Evidence-Based and Clinical Research Laboratory, Department of Health, Social, and Clinical Pharmacy, College of Pharmacy, Chung-Ang University, Seoul 06974, Republic of Korea; 4 Regulatory Science Policy, Pharmaceutical Regulatory Sciences, Chung-Ang University, Seoul 06974, Republic of Korea

**Keywords:** pandemic, emerging infectious disease, vaccine platform technologies, global vaccine organization, vaccine support program

## Abstract

**Background:** The emergence of more than 40 new infectious diseases since the 1980s has emerged as a serious global health concern, many of which are zoonotic. In response, many international organizations, including the US Centers for Disease Control and Prevention (CDC), the World Health Organization (WHO), and the European Center for Disease Prevention and Control (ECDC), have developed strategies to combat these health threats. The need for rapid vaccine development has been highlighted by Coronavirus disease 2019 (COVID-19), and mRNA technology has shown promise as a platform. While the acceleration of vaccine development has been successful, concerns have been raised about the technical limits, safety, supply, and distribution of vaccines. **Objective:** This study analyzes the status of vaccine platform development in global pandemics and explores ways to respond to future pandemic crises through an overview of the roles of international organizations and their support programs. It examines the key roles and partnerships of international organizations such as the World Health Organization (WHO), vaccine research and development expertise of the Coalition for Epidemic Preparedness Innovations (CEPI), control of the vaccine supply chain and distribution by the Global Alliance for Vaccines and Immunization (GAVI), and technology transfer capabilities of the International Vaccine Institute (IVI) in supporting the development, production, and supply of vaccine platform technologies for pandemic priority diseases announced by WHO and CEPI and analyzes their vaccine support programs and policies to identify effective ways to rapidly respond to future pandemics caused by emerging infectious diseases. **Methods**: This study focused on vaccine platform technology and the key roles of international organizations in the pandemic crisis. Literature data on vaccine platform development was collected, compared, and analyzed through national and international literature data search sites, referring to articles, journals, research reports, publications, books, guidelines, clinical trial data, and related reports. In addition, the websites of international vaccine support organizations, such as WHO, CEPI, GAVI, and IVI, were used to examine vaccine support projects, initiatives, and collaborations through literature reviews and case study methods. **Results**: The COVID-19 pandemic brought focus on the necessity for developing innovative vaccine platforms. Despite initial concerns, the swift integration of cutting-edge development technologies, mass production capabilities, and global collaboration have made messenger RNA (mRNA) vaccines a game-changing technology. As a result of the successful application of novel vaccine platforms, it is important to address the remaining challenges, including technical limits, safety concerns, and equitable global distribution. To achieve this, it is essential to review the regulatory, policy, and support initiatives that have been implemented in response to the COVID-19 pandemic, with particular emphasis on the key stages of vaccine development, production, and distribution, to prepare for future pandemics. An analysis of the status of vaccine development for priority pandemic diseases implies the need for balanced vaccine platform development. Also, international organizations such as WHO, CEPI, GAVI, and IVI play key roles in pandemic preparedness and the development and distribution of preventive vaccines. These organizations collaborated to improve accessibility to vaccines, strengthen the global response to infectious diseases, and address global health issues. The COVID-19 pandemic response demonstrates how the synergistic collaboration of WHO’s standardized guidelines, CEPI’s vaccine research and development expertise, GAVI’s control of the vaccine supply chain and distribution, and IVI’s technology transfer capabilities can be united to create a successful process for vaccine development and distribution. **Conclusions**: In preparation for future pandemics, a balanced vaccine platform development is essential. It should include a balanced investment in both novel technologies such as mRNA and viral vector-based vaccines and traditional platforms. The goal is to develop vaccine platform technologies that can be applied to emerging infectious diseases efficiently and increase manufacturing and distribution capabilities for future pandemics. Moreover, international vaccine support organizations should play key roles in setting the direction of global networking and preparing for international vaccine support programs to address the limitations of previous pandemic responses. As a result, by transforming future pandemic threats from unpredictable crises to surmountable challenges, it is expected to strengthen global health systems and reduce the social and economic burden of emerging infectious diseases in the long term.

## 1. Introduction

Since the 1980s, over 40 new and variant infectious diseases have emerged, including Severe Acute Respiratory Syndrome (SARS) in 2003, H1N1/09 influenza in 2009, Middle East Respiratory Syndrome (MERS) in 2012, and Coronavirus Disease 2019 (COVID-19) in 2019 [1,2,3]. Notably, the majority of significant infectious diseases since 2000 have been zoonotic in origin [3]. Therefore, recognizing that 60–80% of these new and variant infectious diseases originated from animals remains crucial [4]. The rapid spread of these pathogens has been facilitated by increased human mobility, climate change, and aging populations, giving rise to concerns about potential pandemics [5]. In 2018, the World Health Organization (WHO) designated potential future pathogens and viruses as “Disease X”, highlighting the importance of preparedness against Emerging Infectious Diseases (EIDs) [3,6].

Epidemic and Pandemic outbreaks since the 20th century and vaccine development timeframes are shown in Table 1 [7]. According to the initial vaccine development period, in the case of influenza, it required a long time to develop an initial vaccine, but since then, the vaccine development time has been much shorter even when new strains have emerged. Furthermore, an initial vaccine for Ebola virus took a long time to develop, but it has a high prevention rate and is still recommended by the WHO [8]. Considering the vaccine development timeframes for previous pandemic diseases, the development of the COVID-19 vaccine was remarkably accelerated despite the fact that no vaccine was previously available.

The coronavirus disease 2019 (COVID-19), which broke out in late 2019 and was declared a pandemic by the WHO in May 2020, is unlike any war, terrorism, or disease the world has ever known. The pandemic has exposed limitations in the ability to diagnose, test, and treat patients by the global healthcare systems. Given the health crisis and economic impact of the pandemic, the development, procurement, and distribution of preventive vaccines are emerging as critical elements for mitigating potential damage.

In response to the COVID-19 pandemic, COVID-19 Vaccines Global Access (COVAX) was launched as a multilateral effort co-led by GAVI, CEPI, WHO, and the United Nations International Children’s Emergency Fund (UNICEF) from 2020 to 2023. The COVAX initiative was established with the objectives of expediting COVID-19 vaccine production and ensuring global equity in vaccine distribution. This initiative has provided nearly two billion doses of vaccines. Although there are still some gaps that need to be supplemented, COVAX was the vaccine pillar of the Access to COVID-19 Tools (ACT) Accelerator. The ACT Accelerator is an innovative global partnership aimed at expediting the discovery, manufacturing, and equitable distribution of COVID-19 Diagnostics tools, treatments, and vaccines [13].

In the example above, the ability to rapidly produce and distribute effective vaccines is key to pandemic preparedness and response strategies. Therefore, vaccine technology platforms and international networks are expected to play crucial roles in controlling the spread of infectious diseases and responding swiftly to the emergence of new or variant viruses in the next generation [14]. Global collaboration is necessary to create a fair platform that provides equal benefits to all countries through safe, effective, and quality-assured vaccines and medicines. International organizations such as the WHO, CEPI, GAVI, Bill & Melinda Gates Foundation (BMGF), Research Investment for Global Health Technology (RIGHT) Foundation, and IVI have actively announced support strategies to establish rapid vaccine development platforms and strategies in response to future pandemics caused by Disease X. In this study, WHO, CEPI, GAVI, and IVI were selected as international vaccine development support organizations that play a coordinated role in vaccine support, and their respective infectious disease preparedness programs were analyzed [15].

This study aims to analyze the ways for efficient and rapid vaccine development, production, and distribution in future pandemics caused by EIDs by examining the key roles and vaccine support programs of international organizations such as WHO, CEPI, GAVI, and IVI through a step-by-step analysis of the vaccine industry value chain. Accordingly, this study analyzes the current status of preventive vaccine platform development and the key roles of international organizations such as WHO, CEPI, GAVI, and IVI in the global pandemic crisis to find ways to prepare for and respond to future pandemics.

The first part analyzes the development of preventive vaccine platforms against the priority pathogens announced by WHO and CEPI, and the second part analyzes key roles and collaborative relationships between international organizations for pandemic response, as well as the distinctive vaccine support programs of each organization, to examine the establishment of global networks for future pandemics, and to consider important issues and implications.

## 2. Materials and Methods

The study collected, analyzed, and reprocessed data using the snowball sampling method [16]. This study was conducted in two main parts. In the first section, we examined the current status of new infectious disease crises both domestically and internationally, as well as trends in vaccine development. The status of infectious disease outbreaks was reconstructed by collecting data on pandemic alert levels announced by the WHO and historical epidemic and pandemic occurrences from articles, journals, research reports, publications, books, and official regulatory guidelines and related reports. The latest vaccine development platform and the main content were compiled through direct review. By examining trends, classifying vaccine development platforms, and reviewing the latest technological trends, we established the research direction. In particular, we organized common diseases expected to cause future pandemics through the pandemic priority disease list presented by WHO and CEPI and collected the current status of vaccine development and the support status of international organizations at each development stage from Clinicaltrials.gov.

In the second section, we collected and analyzed data directly on the support programs of international vaccine support organizations such as WHO, CEPI, GAVI, and IVI from the open webpages of each organization, focusing on the common or distinct roles of each organization, and created diagrams to differentiate between their common and individual roles.

### 2.1. Study Searching

This study focused on the development of preventive vaccine platforms and the role of international organizations during a global pandemic crisis. Literature data on EIDs and vaccines was collected, compared, and analyzed by referring to articles, journals, research reports, publications, books, and official regulatory guidelines and related reports through database platforms such as Clinicaltrials.gov, DBpia, Embase, Google Scholar, KISS, PubMed, Research GATE, Science Direct, Springer, and RISS. In addition, websites of international vaccine-related organizations such as WHO, CEPI, GAVI, and IVI were used to investigate the roles and vaccine support programs through a literature review. In addition, the initiatives and cooperations among the organizations were analyzed by case study methods. Websites of international vaccine organizations and national institutes are listed in Table 2.

#### 2.1.1. Data Search Keywords

Search keywords were applied in varied combinations, including “infectious disease preparedness”, “preventive vaccines for infectious diseases”, “priority pathogens”, “vaccine technology platforms”, “post-pandemic”, “global vaccine support”, “international vaccine organizations”, and “vaccine support programs”. The literature data publication period was set from October 2019 to June 2024, after the outbreak of COVID-19, to collect updated data. The data were not used immediately after collection; instead, the data were modified, translated, and reprocessed to fit the research content.

#### 2.1.2. Comparison of Global Vaccine Support Organizations

In this study, we selected the four international vaccine support organizations presented in the 2023 Vaccine Industry Trends issued by the Ministry of Food and Drug Safety (MFDS) and the Korea Biomedicine Industry Association (KOBIA) [15]. The vaccine support programs of international organizations such as the WHO, CEPI, GAVI, and IVI, which have specialized areas in pandemic response, were divided into categories, including Prevention and Control of Infectious Diseases, R&D for Infectious Disease and Vaccine Technology, Manufacturing and Supply of Vaccines, Pandemic Preparedness, and Establishing a Global Partnership. The contents of each category were extracted and compared. The results of this study are presented in a comparison table to analyze the major vaccine development support programs by category in Appendix A.

## 3. Results

### 3.1. Emerging Infectious Disease Preparedness and Trends of Vaccine Development

#### 3.1.1. Alert Phases of Infectious Disease and Historical Outbreaks

**Figure 1:**
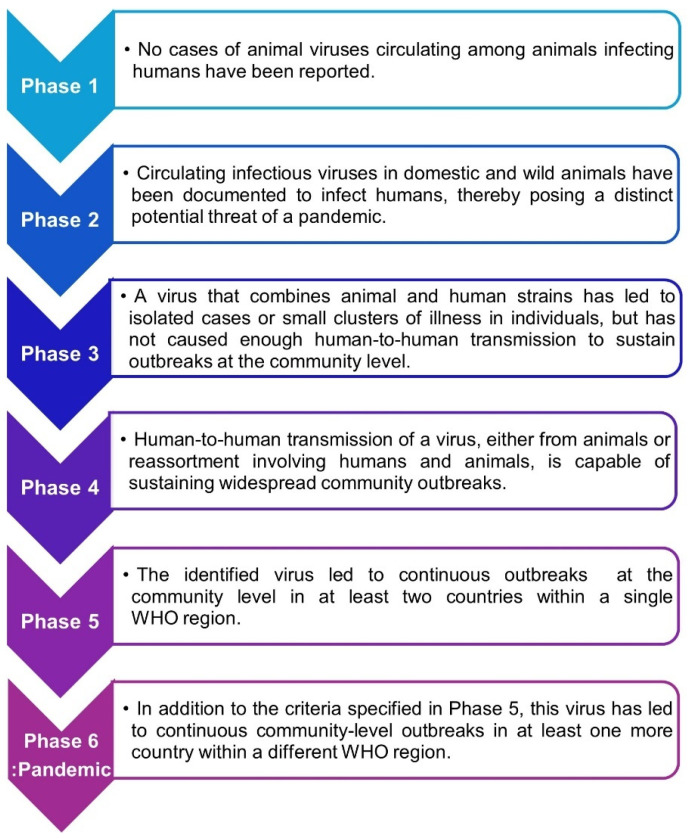
Alert phases of a pandemic outbreak [6].

The WHO classified alert levels from Phase 1 to Phase 6 based on the risk of infectious diseases, defining Phase 6, the highest alert level, as a “pandemic”. The infectious disease alert phases announced by the WHO are shown in Figure 1.

The initial three phases of pandemic alerts concern preparations for a widespread disease, whereas the following three phases require active responses to the crisis. According to the WHO guidelines, Phase 4 is defined as an “epidemic”, and Phase 6 is defined as a “pandemic” worldwide. After Phase 4, countries reinforce precautions to prevent the spread of the disease, such as travel restrictions and increased countermeasures. This phased approach offers an essential direction for efficient responses, given the trends in disease prevalence.

#### 3.1.2. Development of Vaccine Platforms

Vaccine technologies have advanced from traditional vaccine platforms to novel vaccine platforms such as viral vector vaccines and mRNA, each of which has its advantages and disadvantages in terms of immunogenicity, persistency, safety, delivery efficiency, stability, compatibility, and rapid and mass production response to EIDs, as shown in Table 3.

Therefore, a balanced advancement of existing and next-generation technologies is needed to address disease characteristics [17].

• mRNA vaccines

mRNA vaccines have been proposed as a new strategy to provide immunity by delivering mRNAs encoding viral antigens such as the SARS-CoV-2 spike protein, rather than injecting the protein directly. This method improves the efficiency of vaccine production and delivery by using distinct human protein synthesis mechanisms to generate multiple proteins from a single mRNA molecule. Nucleic acids are pivotal components that allow the swift modification of genetic data, enabling faster and easier examination of various antigens [21]. In addition, vaccines based on nucleic acids exhibit novel immunostimulant properties through interactions with innate immune receptors, which may not require additional adjuvants typically required in traditional vaccine formulations. These features make the development and mass production processes more efficient. Theoretically, excessive immune responses in some cases can lead to serious side effects; however, scientific evidence suggests that the risks are not significant compared with traditional vaccine platforms.

One of the key features of mRNA vaccines is that they can be synthesized rapidly and require only the nucleotide sequences of the target viruses. This enables a swift response to novel viruses or strains, which is an important countermeasure against EIDs. Moreover, because no live pathogens are involved in the manufacturing process, mRNA vaccines are much safer and can be manufactured at low cost in small-scale Good Manufacturing Practices facilities [17]. In addition, in the event of a viral mutation, only a limited portion of the genetic material needs to be modified, enabling the development of a novel or supplementary vaccine within a six-week timeframe, much faster than traditional vaccine production. mRNA vaccines have also demonstrated significant efficacy compared to traditional platforms [22]. However, they contain lipids, which result in a relatively short shelf life of approximately 2–3 years, and they require storage at ultra-low temperatures, typically up to −80 °C, making them less favorable in terms of storage and logistics costs compared to plasmid DNA vaccines [23]. The limitations and research progress of mRNA vaccine technologies are summarized in Table 4.

• Next generation: digital vaccine

A digital vaccine is a method of globally sharing genetic information comprising vaccine target antigens via the Internet, enabling the development of self-sufficient vaccinations in vaccine manufacturing facilities. Digital vaccinations have the advantage of not relying on components obtained from live pathogens [28].

The COVID-19 mRNA vaccine serves as a good example of a digital vaccine [29]. In 2020, the Chinese Center for Disease Control and Prevention shared the genome sequence of SARS-CoV-2 online, and hundreds of laboratories around the world used synthetic SARS-CoV-2 genes that encode the spike protein to develop mRNA vaccines without the SARS-CoV-2 virus [30]. The mRNA vaccine developed by Moderna began clinical testing approximately 60 days after the genetic information for SARS-CoV-2 was made public, and the U.S. Food and Drug Administration granted emergency use authorization to the mRNA vaccines developed by Moderna and Pfizer-BioNTech 10 months later. In this approach, digital vaccines not only delivered vital immunizations swiftly to patients during the pandemic but also ushered in a revolution in vaccine manufacturing [31].

In addition, digital vaccines use artificial intelligence (AI) techniques to analyze vaccine candidates and collect data, such as sequencing, through the computational analysis of pathogen genes. Clinical trials should be conducted to collect data on the potential side effects of vaccines based on different patient characteristics (age, sex, and underlying medical conditions). Additionally, the theoretical design and production of vaccines can be completely automated, and remote guidance can be provided. Furthermore, a global network of hospitals for vaccine distribution allows for a data-driven evaluation of production and distribution factors, which can have a positive effect on manufacturing and supply. It can also reduce the burden on healthcare systems by balancing supply and demand. Overall, AI can accelerate the development process, reduce costs, and significantly improve efficiency [32].

#### 3.1.3. Prioritized Infectious Diseases Expected to Cause Future Pandemics

The 26 families of viruses known to infect humans, including influenza viruses and coronaviruses, have caused five pandemics since 1900. The WHO maintains a list of prioritized diseases for pandemics that is updated by a panel of more than 300 scientists every year. The list prioritizes viruses for research and development based on several key factors, including human transmissibility, availability of countermeasures, severity, zoonotic potential, and societal impact. It aims to strengthen preparedness for future pandemics by focusing on global efforts regarding the most pressing viral threats.

When preparing for Disease X, distinguishing between known and unknown diseases is imperative. In the context of Disease X, “X” represents the entirety of an unknown. However, knowledge regarding these emerging diseases is lacking. These diseases can be lethal upon their initial emergence, are highly contagious, and pose a significant threat to our way of life. In addition, when and how these pathogens cross borders or infect individuals remains unknown. What is known is that the next Disease X is approaching, and preparations must be made. The listed diseases in Table 5 represent priority pathogens for the prevention of future pandemics, as outlined in reports published by the WHO and CEPI.

As summarized in Table 6, this study examined vaccines currently in development for priority diseases, excluding those for which vaccines have already been approved and focusing on those still in development. Various types of vaccines, ranging from live-attenuated vaccines to RNA vaccines, are being developed to treat pandemic-priority diseases. Notably, vector vaccines, RNA vaccines, and DNA vaccine platforms are actively being developed. Platform technologies that integrate technologies, such as those for “Disease X” vaccines, are being developed to address a wide range of viruses. International organizations provide support for preventive vaccines against priority diseases in terms of technology and funding. Research and development are being conducted at various scales by laboratories, companies, institutions, and governments. Clinical trials are actively conducted in regions vulnerable to infection risk and in areas with abundant funding and research personnel.

### 3.2. Key Roles and Vaccine Support Programs of International Organizations for Future Pandemics

**Figure 2:**
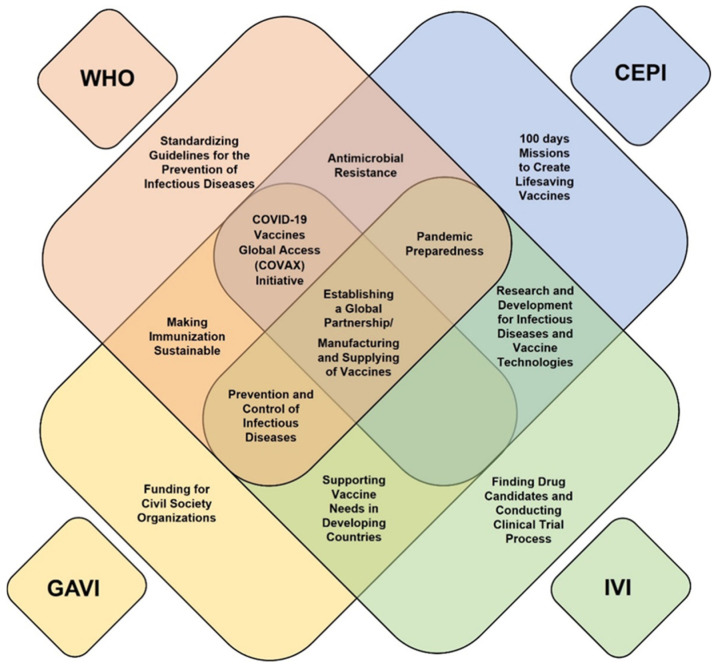
Key roles of global vaccine support organizations: WHO, CEPI, GAVI, and IVI.

Global collaboration is necessary for creating a fair platform that provides equal benefits to all countries through safe, effective, and quality-assured vaccines and medicines. In this study, the WHO, CEPI, GAVI, and IVI were selected as the key international vaccine development support organizations that play a coordinated role in vaccine support, and their respective infectious disease preparedness programs were analyzed. The main roles of these four international organizations in responding to infectious diseases are depicted in Table 7 and Figure 2. In addition, details of ongoing vaccine support programs by international organizations are provided in Appendix A.

## 4. Discussion

### 4.1. Emerging Infectious Diseases Preparedness and Trends of Vaccine Development

The COVID-19 pandemic has demonstrated the capability and efficacy of rapid mass production using novel vaccine platforms. This has contributed to the establishment of platforms capable of rapidly responding to pandemic-priority pathogens and preventing global health issues in future pandemics. While new technologies such as mRNA and vector vaccines have gained prominence, balancing the development of traditional vaccines and bridging technology gaps remains imperative. These technological advancements will enable the construction of a robust health system capable of preventing Disease X and ensuring a safe global health environment [21]. Numerous platform technologies have been applied to COVID-19 vaccines, and mRNA vaccines have emerged as next-generation technologies that feature rapid development and mass production capabilities. However, additional obstacles have arisen regarding safety and limits in storage and supply, such as the need for low temperatures to maintain stability and the short shelf life of the products. Technological research, including vaccine delivery vehicles, lipid nanoparticle formulations, and adjuvants, has overcome these obstacles and accelerated the development of vaccine platforms.

Governments, regulatory authorities, related agencies, and international organizations have quickly analyzed and addressed the needs at all stages of vaccine development. The establishment of swift projects, financial support, and global cooperation helped reduce the impact of the pandemic from the initial research and clinical stages to the manufacturing, licensing, and distribution stages.

To prepare for the next pandemic, various types of vaccine platforms, ranging from live-attenuated vaccines to RNA vaccines, are being developed. Additionally, vaccines that can prevent the spread of multiple viruses are currently being researched. International organizations provide technical and financial support for preventive vaccines against priority pandemic diseases, with research and development involving a range of scales, from laboratories to governments. According to the status of their clinical studies, these vaccines are being actively used in regions with populations vulnerable to infections, as well as in areas with abundant funding and expertise. It aims to ensure that vaccines are effective in the most vulnerable populations while also deploying available resources. In this regard, the global preparedness and response capabilities for future health threats can be improved by identifying effective regulatory options and strategies for vaccine platform development.

### 4.2. Key Roles and Vaccine Support Programs of International Organizations for Future Pandemics

The COVID-19 pandemic has intensified international efforts and competition to accelerate vaccine development. The success of mRNA vaccines from companies such as Pfizer and Moderna, as well as numerous vaccines developed in the US and EU, indicates a high growth potential for the vaccine market. However, the high costs associated with large-scale clinical trials and concerns about demand reduction during the post-pandemic period may lead to market monopolization by global companies. This suggests that the role and cooperation of international organizations are more important than ever for equitable vaccine distribution and efficient vaccine development.

The WHO, CEPI, GAVI, and IVI play key roles in global vaccine platform development and global vaccine distribution. The WHO leads global health response coordination, provides policy and standard guidelines, and operates the prequalification program to evaluate vaccine safety and efficacy. CEPI focuses on supporting vaccine platform development and research for EIDs through its properties and partnerships for research and support programs. GAVI concentrates on equitable vaccine distribution and procurement and provides support by utilizing its joint purchasing program to reduce costs and improve accessibility. The IVI specializes in researching candidates, technology transfer, and production infrastructure support for developing countries, focusing on advancing vaccine research, development, and production capabilities of developing countries. To improve pandemic preparedness and response, organizations should strengthen their collaboration and global networking, including information sharing, integrated development strategies, and cooperation in resource mobilization and funding support. Therefore, global collaboration can prevent duplicate investments and ensure efficient use of limited resources.

The integration of the specialized strengths and expertise of these organizations, such as the policy support of the WHO, research and development capabilities of the CEPI, supply chain management of the GAVI, and technology transfer expertise of the IVI, can establish an effective vaccine development and distribution process. These efforts could improve vaccine accessibility in developing countries, strengthen vaccine development and manufacturing capacities, and reduce the burden of unpredictable infectious diseases.

## 5. Conclusions

The COVID-19 pandemic has dramatically changed the global health landscape, highlighting the importance of rapid vaccine development and equitable distribution to mitigate health, economic, and social impacts. To prepare for future pandemics, a diverse approach to vaccine platform development is essential. This should include a balanced investment in both novel technologies, such as mRNA- and viral vector-based vaccines, and traditional platforms. The goal is to develop vaccine platform technologies that can be efficiently applied to emerging infectious diseases, thereby increasing the manufacturing and distribution capabilities for future pandemics. Moreover, international vaccine support organizations should play a key role in setting the direction of global networking and preparing international vaccine support programs to address the limitations of previous pandemic responses. Therefore, transforming future pandemic threats from unpredictable crises to surmountable challenges is expected to strengthen global health systems and reduce the social and economic burdens of emerging infectious diseases in the long term.

## Figures and Tables

**Table 1:** Epidemic and pandemic outbreaks since the 20th century and vaccine development timeframes.

Classification	Year of Outbreak	Disease	Subtype	Reproduction Number (r_0_ ^1^)	Fatality Rate (%)	Initial Vaccine Development Period ^2^
**Epidemics ^3^**	1977	Russian Influenza A	H1N1	-	-	1942
	1997	Avian Influenza	H5N1	1.3–1.6	>50	1997–2009
	2002	Severe Acute Respiratory Syndrome (SARS)	SARS-CoV	1.5	>9.5	2003–present
	2003	Avian Influenza	H5N1	1.3–1.6	>50	1997–2009
	2012	Middle East Respiratory Syndrome (MERS)	MERS-CoV	0.4–0.9	20–40	2012–present
	2013	Influenza A virus	H7N9	0.27	>38	March 2013–October 2013
	2014	Ebola virus	ZEBOV	1.56–1.9	>50–90	1976–2019
**Pandemics**	1918	Spanish Influenza	H1N1	1.47–2.27	>2–3	1917–1942
	1957	Asian Influenza	H2N2	1.53–1.70	<0.2	February 1957–June 1957
	1968	Hong Kong Influenza	H3N2	1.56–1.85	<0.2	July 1968–November 1968
	2009	Swine Influenza	H1N1/09	1.30–1.70	0.1	April 2009–September 2009
	2019	Severe Acute Respiratory Syndrome Coronavirus 2 (SARS-CoV-2, COVID-19)	SARS-CoV-2	2.2–3.3	0.995	December 2019–November 2020

^1^ The reproduction number (r_0_) is the reproduction index when an infectious disease occurs in a population that is not immune to the disease (emerging, unvaccinated) and control interventions have not yet been introduced. r_0_ is not a unique value given for each infectious disease, but it is generally believed to be in a similar range for the same infectious disease [9]. ^2^ Timeframe for the first vaccine development after the disease outbreak. ^3^ An epidemic is a sudden and unexpected outbreak of a disease in a population in a specific geographic area, and a pandemic is when a disease spreads across multiple countries and affects many people. Reproduction number and fatality rate of diseases vary depending on the data sources. Data Source: Compiled from the Infectious Disease Portal of the Centers for Disease Control and Prevention and media reports [10,11,12].

**Table 2:** Websites of international vaccine organizations and national institutes.

Organization/Institute	Website
World Health Organization (WHO)	https://www.who.int/ (accessed on 7 January 2025)
Coalition for Epidemic Preparedness Innovations (CEPI)	https://cepi.net/ (accessed on 7 January 2025)
Global Alliance for Vaccines and Immunization (GAVI)	https://www.gavi.org/ (accessed on 7 January 2025)
International Vaccine Institute (IVI)	https://www.ivi.int/ (accessed on 7 January 2025)
Ministry of Food and Drug Safety (MFDS)	http://www.mfds.go.kr/index.do (accessed on 7 January 2025)
Food and Drug Administration (FDA)	http://www.fda.gov (accessed on 7 January 2025)
European Medicines Agency (EMA)	https://www.ema.europa.eu/ (accessed on 7 January 2025)

**Table 3:** Classification and characteristics of vaccine platforms [17,18,19,20].

Generation	Platform		Drug Effectiveness				Productivity			
			Immunogenicity	Persistency	In-Vivo Safety	Delivery Efficiency	Storage and Stability	Viral Compatibility	Production Safety	Rapid and Mass Production
**1st** **Generation**	Whole Virus Vaccine	Live Attenuated Virus Vaccine	High	High	Low	High	Refrigerated storage	Low	Low	Prolonged culture steps are required to obtain attenuated viral strains
		Inactivated Virus Vaccine	Adjuvants can be used	Low	High	Requires two or more doses to be effective	• Refrigerated storage • Possibility of structural damage to the antigen or epitope due to partial expression	Medium	High	Deploy existing cGMP-grade vaccine production infrastructure
**2nd** **Generation**	Protein- based Vaccine	Protein Subunit Vaccine	Adjuvants can be used	Low	High	• Low • A separate delivery system is required	• Refrigerated storage • Possibility of structural damage to the antigen or epitope due to partial expression	Medium	High	Securing high yield and needs for large-scale cultivation facilities limit production capacity
		Virus-Like Particle Vaccine	High	High	High	An effective immune response can be induced even at low doses	Refrigerated storage	Medium	High	Production speed is not fast enough
**3rd** **Generation**	Nucleic- Acid Vaccine	mRNA Vaccine	Adjuvants can be used	Low	High	• Low • A separate delivery system is required	Requires deep freezing due to instability of RNA and LNPs	Medium	High	Enables rapid and Low-cost production in small-scale GMP facilities
		DNA Vaccine	Low	Depending on conditions	High	Low	Room temperature storage	Medium	High	Enables rapid and low-cost production in small-scale GMP facilities
	Viral Vector Vaccine		High	Low	High	Pre-existing immunity to the vector may reduce vaccine efficacy.	Refrigerated storage	High	Medium	Securing high yield and needs for large-scale batch and down-stream process complexity limit production capacity
**Next** **Generation**	Digital Vaccine	• Networking Vaccine, • Artificial Intelligence (AI) Combined Vaccine	Development practices and validations are lacking, thereby requiring improvement and strategy refinement				-	High	High	Designing and manufacturing of vaccines by automated programs

**Table 4:** Limitations and research progress of mRNA technology.

Subject	Limitations	Progress of Development
**Stability**	There are difficulties in transport and management due to the instability of RNA and LNPs, which requires ultra-low temperature freezing [24,25].	• Suzhou Abogen Biosciences in China is engaged in the development of a technology that enhances the quality and purity of lipid nanoparticles (LNPs) and enables storage at refrigerated temperatures (2–8 °C) [26]. • CureVac in Germany is developing a technology to fold RNA into a compact 3D structure to improve low thermal stability [26].
**Dosage and Efficacy**	• A mRNA vaccine requires multiple doses to achieve the same effect as a conventional vaccine. • A separate delivery system is required due to the inefficiency of in vivo delivery [27].	• The mRNA vaccine from Curevac in Germany, currently in Phase 2 clinical trials, is expected to have great potential for further development as it is effective with a relatively small dose [26]. • Vaxess Technologies in the US has developed a skin patch with dissolvable microneedles that can slowly release a vaccine [26].
**Polyvalent Vaccine** **Technology**	Each type of virus requires a redesign, and each type requires a different vaccine [26].	• Universal flu shot ^1^ vaccine, which is effective against various new influenza viruses without redesigning, is being developed [26]. • A vaccine made by mixing four mRNAs encoding different influenza proteins was found to protect against infection by a specific strain of influenza virus in animal experiments, such as those involving mice, at the University of Pennsylvania in the US [26].

^1^ Universal flu shot: vaccination that is effective against all influenza strains regardless of virus mutation, antigenic drift, or antigenic variation.

**Table 5:** Prioritized diseases announced by the World Health Organization (WHO) and the Coalition for Epidemic Preparedness Innovations (CEPI).

No.	Priority Disease [33,34]	Viral Family [35,36]	WHO *	CEPI **	Type of Authorized Vaccines ***
1	COVID-19	Coronaviridae	O	O	Viral Vector, RNA, Inactivated, Subunit, etc.
2	Crimean-Congo Haemorrhagic Fever	Bunyaviridae	O	-	None
3	Ebola Virus Disease	Filoviridae	O	O	Viral Vector
4	Marburg Virus Disease	Filoviridae	O	-	None
5	Lassa Fever	Arenaviridae	O	O	None
6	Middle East Respiratory Syndrome Coronavirus (MERS-CoV)	Coronaviridae	O	O	None
7	Severe Acute Respiratory Syndrome (SARS)	Coronaviridae	O	-	None
8	Nipah And Henipaviral Diseases	Paramyxoviridae	O	O	None
9	Rift Valley Fever(RVF)	Bunyaviridae	O	O	None
10	Zika	Flaviviridae	O	-	None
11	Chikungunya	Togaviridae	-	O	Live-attenuated
12	Disease X ^1^	-	O	O	None

* The latest priority diseases announced in February 2018 by the WHO are marked with “O”. ** The latest priority diseases announced in January 2019 by CEPI are marked with “O”. *** Diseases for which there are no authorized vaccines have been marked as “None”. ^1^ The potential for a serious international pandemic to be caused by a pathogen currently unknown to cause human disease is represented by “Disease X”.

**Table 6:** Status of vaccine development for priority diseases that do not have authorized vaccines yet.

Viral Family	Priority Disease	Platform	Candidate ^1^	Developer	Collaborator ^2^	Phase	Location
Bunyaviridae	**Crimean-Congo Haemorrhagic Fever**	Viral Vector	ChAdOx2 CCHF [37]	University of Oxford	-	Phase 1	United Kingdom
		Inactivated	KIRIM-KONGO-VAX [38]	The Scientific and Technological Research Council of Turkey	Monitor CRO	Phase 1	Turkey
	**Rift Valley Fever (RVF)**	Viral Vector	ChAdOx1 RVF [39]	University of Oxford	MRC/UVRI and LSHTM Uganda Research Unit	Phase 1	Uganda
		Live attenuated	DDVax [40]	UC Davis	**CEPI** ^3^	Preclinical	-
			hRVFV-4s [41]	Wageningen University		Phase 1	Belgium
			TSI-GSD-223 [42]	U.S Army Medical Research and Development Command	-	Phase 2	USA
		Inactivated	TSI-GSD 200 [43]	U.S Army Medical Research and Development Command	-	Phase 2	USA
Filoviridae	**Marburg Virus Disease**	Viral Vector	cAd3-Marburg vaccine [44]	Albert B. Sabin Vaccine Institute	Biomedical Advanced Research and Development Authority	Phase 2	Uganda, Kenya
			ChAdOx1 Marburg [45]	University of Oxford	Department of Health and Social Care as part of the UK Vaccine Network (UKVN)	Phase 1	United Kingdom
			PHV01 [46]	Public Health Vaccines LLC	Biomedical Advanced Research and Development Authority	Phase 1	USA
Arenaviridae	**Lassa Fever**	Viral Vector	ChAdOx1-Lassa-GPC [47]	University of Oxford	**CEPI**	Preclinical	-
			rVSV∆G-LASV-GPC [48]	International AIDS Vaccine Initiative (IAVI)		Phase 2	Ghana, Liberia, Nigeria
			EBS-LASV [49]	Emergent BioSolutions		Phase 1	Ghana
			MV-LASV [50]	Themis Bioscience GmbH		Phase 1	Belgium
		DNA	INO-4500 [51]	Inovio Pharmaceuticals		Phase 1	USA
Coronaviridae	**Middle East Respiratory Syndrome Coronavirus (MERS-CoV)**	Viral Vector	ChAdOx1 MERS [52]	University of Oxford, Barinthus	**CEPI**	Phase 1	United Kingdom
			MVA-MERS-S [53]	IDT Biologika GmbH		Phase 1	Germany, The Netherlands
			ChAdOx1 MERS [54]	King Abdullah International Medical Research Center	University of Oxford	Phase 1	Saudi Arabia
			BVRS-GamVac [55]	Gamaleya Research Institute of Epidemiology and Microbiology	Acellena Contract Drug Research and Development	Phase 1/ Phase 2	Russian Federation
			MVA-MERS-S_DF-1 [56]	Universitätsklinikum Hamburg-Eppendorf	**CEPI**	Phase 1	Germany, The Netherlands
		DNA	INO-4700 [57]	Inovio Pharmaceuticals		Phase 2	Jordan, Kenya
Paramyxoviridae	**Nipah and Henipaviral Diseases**	Viral Vector	ChAdOx1 NipahB [58]	University of Oxford	**CEPI**	Phase 1	United Kingdom
			PHV02 [59]	Public Health Vaccines LLC		Phase 1	USA
		Protein based	HeV-sG-V [60]	Auro Vaccines and PATH		Phase 1	USA
		RNA	mRNA -1215 [61]	National Institute of Allergy and Infectious Diseases (NIAID)	Moderna TX, Inc	Phase 1	USA
Flaviviridae	**Zika**	Inactivated	Zika Virus Purified Inactivated Vaccine (ZPIV) [62]	National Institute of Allergy and Infectious Diseases (NIAID)	-	Phase 1	USA
			VLA1601 [63]	Valneva Austria GmbH	Emergent BioSolutions	Phase 1	USA
			Purified Inactivated Zika Virus Vaccine (PIZV) [64]	Takeda	-	Phase 1	USA
		DNA	VRC-ZKADNA085-00-VP [65]	National Institute of Allergy and Infectious Diseases (NIAID)	-	Phase 2	USA
			GLS-5700 [66]	GeneOne Life Science, Inc.	Inovio Pharmaceuticals	Phase 1	Puerto Rico
		Live-attenuated	rZIKV/D4Δ30-713 [67]	National Institute of Allergy and Infectious Diseases (NIAID)	-	Phase 1	USA
		RNA	mRNA-1893 [68]	Moderna TX, Inc.	Biomedical Advanced Research and Development Authority	Phase 2	USA
			mRNA-1325 [69]			Phase 1	
		Viral Vector	ChAdOx1 Zika [70]	University of Oxford	-	Phase 1	United Kingdom
			MV-ZIKA [71]	Themis Bioscience GmbH	-	Phase 1	Austria
**Disease X** ^4^ [34]		RNA	Undisclosed	Akagera Medicines	**CEPI**	Preclinical	-
			Undisclosed	Amplitude Therapeutics		Preclinical	-
			Undisclosed	BioNTech		Phase 1	-
			Undisclosed	Celestial Therapeutics		Preclinical	-
			Undisclosed	Chungbuk National University		Preclinical	-
			Undisclosed	Curevac		Preclinical	-
			Undisclosed	Emervac		Preclinical	-
			Undisclosed	Genova		Preclinical	-
			Undisclosed	HMRI		Preclinical	-
			Undisclosed	Imperial College London		Preclinical	-
			Undisclosed	Lemonex Inc		Phase 1	South Korea
			Undisclosed	Moderna TX, Inc.		Preclinical	-
			Undisclosed	SK Bioscience		Preclinical	-
			Undisclosed	Tiba bio		Preclinical	-
		Viral Vector	Undisclosed	University of Oxford		Preclinical	
		Protein based	Undisclosed	University of Queensland		Preclinical	

^1^ The name of the candidate vaccine in development as published on Clinicaltrial.gov and in the media. ^2^ If the collaborator is an international organization that is being studied in this research, we have marked it in **bold**. ^3^ Coalition for Epidemic Preparedness Innovations. ^4^ “Disease X” represents the knowledge that a serious international pandemic could be caused by a pathogen currently unknown to cause human disease. The R&D Blueprint explicitly seeks to enable early cross-cutting R&D preparedness that is also relevant for an unknown Disease X.

**Table 7:** Key features of the four organizations.

Organization	Year and Entity of Foundation	Key Roles and Strengths	Support Target	Operating Method	Roles in Vaccine Development Stages
**WHO**	1948, United Nations (UN)	➀ Response and coordination in global health crises ➁ Policy and technical standardization ➂ Vaccine Prequalification (PQ) program	Worldwide, including Member Countries	Presenting vaccine roadmaps and policy guidelines in collaboration with governments around the world	Early stages of vaccine development and distribution stages through establishing a vaccination strategy and roadmap
**CEPI**	2017, International Coalition	➀ Funding and accelerating vaccine research ➁ Development technologies for emerging infectious diseases	Areas with Emerging Infectious Disease Threats	Directly funding vaccine development companies or research institutions to promote vaccine development.	Vaccine research and development in emerging infectious diseases; early stages of vaccine development to prepare for mass production promoting
**GAVI**	2000, United Nations (UN)	➀ Management of the vaccine supply chain ➁ Distribution vaccine worldwide	Developing Countries	Global vaccine procurement; vaccine supply in developing countries	Global vaccine distribution and vaccination project management
**IVI**	1997, United Nations Development Program (UNDP)	➀ Development and transferring vaccine technologies ➁ Supporting the research and production capabilities of developing countries	Developing Countries	Conducting a vaccine development project and operating technology transfer and training programs for developing countries	Research for candidates and technology transfer for developing countries

## Data Availability

All data presented in the study are available from the corresponding author upon request.

## Appendix A

**Table A1:** Comparison Table of Major Vaccine Support Programs from WHO, CEPI, GAVI, and IVI ^1^.

Subject	WHO	CEPI	GAVI	IVI
**I.** **Prevention and Control of Infectious Diseases**	**➀** **Infection Prevention and Control (IPC):** Global action plan and monitoring framework on Infection prevention and control (IPC), 2024–2030 Establishes standardized guidelines for the prevention of infectious diseases and provides technical assistance for the development of IPC policies and guides. **➁** **Global Vaccine Action Plan (GVAP):** WHO provides vaccines against more than 20 life-threatening diseases, including diphtheria, tetanus, pertussis, whooping cough, flu, and measles, saving 3.5 to 5 million lives each year.		**➀** **Improving Health System and Immunization Strengthening** GAVI is contributing to strengthening health systems and training health workers in developing countries, thereby improving the capacity of health systems and their ability to respond to health crises. **➁** **Maintaining High Immunization Coverage and Equitable Accessibility of Preventive Vaccines** GAVI has made substantial contributions to global health immunization for over 1 billion children in 78 developing countries, potentially saving over 17.3 million lives. From common pediatric illnesses to emerging diseases such as COVID-19 and Ebola, the organization’s comprehensive strategy tackles a wide range of infectious diseases.	**➀** **Antimicrobial Resistance (AMR)** IVI and its partners are addressing “Antimicrobial resistance (AMR)”, a top global health priority, by improving data quality and quantity, sharing this information with policy establishment, and strengthening quality assurance systems in laboratories to ensure reliable AMR data.
**II.** **R&D for Infectious Disease and Vaccine Technology**	**➀** **Research in Emergencies** Scientific progress through programs that promote international expert collaboration swiftly develops crucial resources such as diagnostic tools, vaccines, and therapeutic interventions, aiming to mitigate the severity of pandemics and infectious disease impacts. **➁** **R&D Blueprint** The R&D Blueprint serves as a comprehensive global strategy and framework, enabling the swift execution of research and development initiatives during health crises. Its principal goal is to facilitate the rapid distribution of efficient diagnostics, immunizations, and therapeutics that can secure lives and avert large-scale health emergencies.	**➀** **Accelerating of Vaccine Technology Development** CEPI focuses on researching innovative technologies that hasten the development of safe, effective, and accessible vaccines to tackle emerging disease outbreaks. **➁** **Supporting the Development of Platform Technologies** CEPI is improving the vaccine platform and manufacturing techniques to rapidly respond to emerging diseases by supporting numerous vaccine platforms and establishing the Global Vaccine Library with prototype vaccines for each viral family. CEPI’s early investments in these technologies and priority-pathogen vaccines are generating critical data for “the 100 Days Mission”, focusing on pandemic potential pathogens and advocating for enhanced coordination and information sharing among R&D funders.		**➀** **Discovery of Promising Candidates** With the assistance of specialist teams in molecular biology and bioinformatics, the IVI has substantial resources and knowledge in the fields of microbiology, immunology, and genomics. **➁** **Vaccine Development and Research** a. IVI’s research portfolio extends over its primary programs for cholera, enteric fever, dengue, and MERS. IVI also investigates hepatitis A and E, human papillomavirus, and tuberculosis. b. IVI’s technology transfer is contingent upon the manufacturer’s ability to produce high-quality, low-cost vaccines in a country with a WHO-approved National Regulatory Authority (NRA). **➂** **Regulatory Review and Approval** IVI supports manufacturers and sponsors of novel vaccine products throughout the regulatory approval process by providing guidance on navigating the regulatory pathway, assisting with steps such as investigating new drug applications, and facilitating manufacturing facility inspections, while also helping them obtain WHO approval to ensure vaccines meet quality, safety, and efficacy standards. **➃** **Vaccine Safety** IVI enhances vaccine safety by aiding health authorities in collecting and analyzing reports of adverse events post-vaccination through the development of the “Vaccine Adverse Events Information Management System (VAEIMS)”.
**III.** **Manufacturing and Supply of Vaccines**	**➀** **COVID-19 Vaccine Global Access, COVAX Initiative** As a co-leader of COVAX, WHO developed a worldwide vaccine distribution strategy and offered overall standard guidelines during the COVID-19 pandemic. In addition, WHO evaluated the efficacy and safety of the vaccines, tracked countries’ vaccine preparedness, and offered guidelines for equitable vaccine distribution. WHO therefore contributed significantly to strengthening COVAX’s ethical and scientific basis.	**➀** **Enabling Equitable Access** CEPI is accelerating vaccine and biological defense development against potential epidemics and pandemics, while actively working to redesign global health preparedness systems for more equitable and effective outcomes through strategic investments, and policy engagements. **➁** **Strengthening research and manufacturing capabilities** CEPI supports activities to strengthen disease surveillance, vaccine manufacturing, research, and clinical trials capacities, thereby improving global preparedness for infectious diseases.	**➀** **COVID-19 Vaccine Global Access, COVAX Initiative** During the COVID-19 pandemic, GAVI played a pivotal role in the operational and financial aspects of COVAX. It directed the design, implementation, and management of the COVAX Facility. GAVI led the large-scale procurement and equitable distribution of vaccines, managing the financial and legal relationships with the 193 participating countries1. GAVI coordinated vaccine support to 92 low-income countries through the COVAX “Advance Manufacturing Commitment (AMC)” and worked with UNICEF and WHO to support country-specific vaccine preparation and distribution.	**➀** **Building Capacity in Vaccines and Vaccination** To foster sustainability and self-sufficiency in vaccines and vaccination in developing countries, we enhance capacity through various means. This includes technology transfer, technical assistance, and support manufacturers, as well as offering technical assistance to “National Regulatory Authorities (NRAs)”.
**IV.** **Pandemic Preparedness**	**➀** **Preparing and Preventing Epidemics and Pandemics** WHO collaborates with a variety of specialists to coordinate global resources and modify plans for regional and national implementation. Plans to prevent the spread of cholera, eradicate yellow fever epidemics, and get prepared for potential influenza pandemics are significant undertakings. WHO also manages global emergencies and coordinates the international coordinating groups on Vaccine Provision, which ensures the rapid distribution of vaccines and antibiotics during major disease outbreaks.	**➀** **Creating Lifesaving Vaccines in 100 Days** The “100 Days Mission” was established by CEPI to prevent the initial spread of viruses and lessen the socioeconomic effects of pandemics by developing and distributing vaccines quickly. **[Three Steps of the “100 Days Mission”]** **Step 1**. Adaptation of known prototype vaccine candidates as an immediate response to develop and manufacture virus-specific vaccines (30 days) **Step 2.** Generating evidence in immediately scaled-up clinical trials (60 days) **Step 3.** When the immunogenicity of a virus-specific vaccine has been confirmed but efficacy has not been provided, an application for emergency authorization for use in populations in the most at-risk areas is filed (10 days) **➁** **Priority-pathogen vaccines** CEPI concentrates to develop vaccines for priority pathogens with epidemic or pandemic potential, having supported over 30 vaccine candidates.		
**V.** **Establishing a Global Partnership**	**➀** **Immunization Agenda 2030 (IA2030)** The main objective of IA2030 is to improve global immunization access and efficacy by 2030 by enhancing roles, responsibilities, and data utilize to encourage action and responsibility. This could potentially save 50 million lives in the world. **➁** **Division of Programs for Disease Control** WHO’s Division of Programs for Disease Control aims to eradicate infectious diseases and improve the control of non-communicable diseases, which include cancer, diabetes, cardiovascular disease, and chronic respiratory disorders, as well as mental health issues. **➂** **The Global Health Emergency Corps (GHEC)** GHEC is a framework meant to strengthen the capability of health emergency workforces, as well as a collaborative platform for countries and health emergency networks, suited to each country’s and region’s distinctive requirements.	**➀** **Global Partnership** For international organizations engaged in many facets of vaccine development and implementation, “The Joint Coordination Group (JCG)” provides a collaborative platform. Throughout the entire vaccine lifecycle, from early research to final administration, this international roundtable unites disparate organizations to enable smooth coordination.	**➀** **Public-Private Partnership** GAVI is a public-private partnership dedicated to saving children’s lives and improving health by enhancing access to immunization in 72 developing countries. WHO, UNICEF, the World Bank, the vaccine industry from both developed and developing regions, research and technical agencies, civil society organizations, the “Bill and Melinda Gates Foundation”, and other private organizations are among the key immunization stakeholders that were brought together by the Alliance.	**➀** **IVI Partnership** IVI collaborates with authorized vaccine manufacturers, primarily from developing countries, to develop vaccines by offering financial and technology transfer assistance for development processes, scale-up, clinical studies, registration, and WHO pre-qualification. IVI is dedicated to developing high-quality vaccines that meet WHO requirements and supplying them in sufficient quantities at affordable prices.

^1^ The contents of the table above are referred from the websites of WHO, CEPI, GAVI, and IVI.
